# Implementation of a pragmatic randomized trial of screening for chronic kidney disease to improve care among non-diabetic hypertensive veterans

**DOI:** 10.1186/s12882-017-0541-6

**Published:** 2017-04-12

**Authors:** Carmen A. Peralta, Martin Frigaard, Anna D. Rubinsky, Leticia Rolon, Lowell Lo, Santhi Voora, Karen Seal, Delphine Tuot, Shirley Chao, Kimberly Lui, Phillip Chiao, Neil Powe, Michael Shlipak

**Affiliations:** 1 San Francisco VA Medical Center, 4150 Clement St., 111A1, San Francisco, CA 94121 USA; 2 University of California, San Francisco, 533 Parnassus Avenue, San Francisco, CA 94117 USA; 3 University of California, San Francisco, 533 Parnassus Ave, UC Hall, San Francisco, CA 94143 USA; 4 San Francisco General Hospital, 1001 Potrero Ave, SFGH 100, San Francisco, CA 94110 USA

**Keywords:** Chronic kidney disease, Blood pressure, Hypertension, Screening

## Abstract

**Background:**

Whether screening for chronic kidney disease (CKD) can improve the care of persons at high risk for complications remains uncertain. We describe the design and early implementation experience of a pilot, cluster-randomized pragmatic trial to evaluate the feasibility, implementation, and effectiveness of a “triple marker” CKD screening program (creatinine, cystatin C and albumin to creatinine ratio) for improving care among hypertensive veterans seen in primary care at one Veterans Administration Hospital.

**Methods/design:**

Non-diabetic hypertensive veterans age 18–80 without known CKD were randomized in clusters determined by primary care provider (unit of randomization) into three arms. Usual care will be compared with two incrementally intensified treatment strategies: (1) screen for CKD followed by patient and provider education or (2) screen-educate plus a clinical pharmacist-led CKD and BP management program. The primary clinical outcome is systolic blood pressure (BP) change from baseline. Secondary clinical outcome is BP control. The primary process outcomes is triple marker screening (across three arms), and secondary process outcomes include use of inhibitors of the renin-angiotensin system (ACE/ARB) overall and in persons with albuminuria, CKD recognition by PCP, use of non-steroidal anti-inflammatory drugs (NSAIDs) and NSAID education by PCP. The design uses the Veterans Health Administration electronic health record (EHR) to identify participants, deliver the interventions and ascertain study outcomes. Assessment of the program implementation will use the Reach, Effectiveness, Adoption, Implementation, and Maintenance (RE-AIM) framework. Study duration is 12 months.

**Results:**

A total of 1,819 patients have been randomized within 41 provider clusters. The median age (interquartile range) is 68 years (61–72), and 99% of participants are male. Approximately 16% are Black, and 5% Hispanic. In the first 6 months of the trial, 434 triple marker screening tests have been ordered, and 217(50%) have been tested. A total of 48 new CKD cases have been identified among those tested, for a preliminary yield of 22%.

**Conclusion:**

We have successfully implemented a pragmatic protocol that uses the EHR to identify and characterize eligible participants, deliver the intervention, and ascertain study outcomes with high rates of participation by providers and patients. Results from this study can guide design of pragmatic trials in the field of CKD.

**Trial registration:**

NCT02059408; Date or Registration: 1/17/2014.

**Electronic supplementary material:**

The online version of this article (doi:10.1186/s12882-017-0541-6) contains supplementary material, which is available to authorized users.

## Background

The value of screening for chronic kidney disease (CKD) remains uncertain. Several lines of evidence suggest that screening may be a cost-effective strategy to reduce the burden of CKD complications in certain high risk groups. For example, CKD affects over 20 million adults in the U.S. and is defined by an estimated glomerular filtration rate (eGFR) <60 ml/min/1.73 m^2^ or an albumin-to-creatinine ratio (ACR) ≥ 30 mg/g, two readily available clinical tests [1, 2]. Persons with CKD are at high risk for complications including cardiovascular events, progression to end stage renal disease (ESRD), hospitalizations, cognitive and functional decline, and premature death [3–6]. Early detection and appropriate classification of CKD makes it possible to optimize treatments for improving blood pressure (BP) control, increase use of renin-angiotensin system inhibitors (ACE or ARB) for persons with proteinuria, and withdraw certain nephrotoxic medications [7]. CKD is largely asymptomatic and it often remains undetected until it has advanced; the vast majority of affected individuals are unaware [8].

Despite its high prevalence, low recognition, high burden of complications, and easy detection, there is no agreement on a national systematic program to screen for CKD in the United States. This is largely due to limited evidence as to whether screening for CKD in high risk persons improves outcomes. To our knowledge, no randomized trials have evaluated the effectiveness of screening for CKD to improve care or to reduce adverse events. Modeling studies have suggested that screening for CKD may be cost-effective among persons with hypertension or diabetes [9–12]. The United States Preventive Services Task Force (USPSTF) has recently graded the effectiveness of CKD screening as “*I”*, insufficient evidence to make a recommendation [13]. Another impediment to assessing the value of CKD screening is that, in practice, despite many adults having serum creatinine measurements, and most laboratories in the U.S. reporting corresponding eGFR, documentation of CKD status in the medical record remains low, suggesting that health care providers may not recognize its presence or importance [14].

Moreover, reliance on serum creatinine alone to determine CKD status misclassifies persons [15]. International guidelines recommend testing for albuminuria, in addition to eGFR, to classify CKD [7]. Our group has shown that cystatin C improves risk stratification of persons across a wide range of creatinine based eGFR estimates [16]. We have shown that a “triple-marker approach” using serum creatinine, cystatin C, and the urine ACR significantly improves CKD detection and risk classification for complications, compared with creatinine alone [15, 17]. We found that persons with eGFRcys <60 ml/min/1.73 m^2^ and eGFRcreat <60 ml/min/1.73 m^2^ and ACR ≥30 mg/g have the highest risk for death and progression to ESRD [15]. Therefore, we believe that the triple marker approach allows for efficient screening and risk stratification concomitantly.

We designed this pilot, cluster-randomized pragmatic trial to evaluate the feasibility, implementation, and effectiveness of a “triple marker” CKD screening approach for improving care among non-diabetic hypertensive veterans seen in primary care at one Veterans Administration Hospital over 12 months. The three arms of our trial will compare usual care to two incrementally intensified treatment strategies: (1) screen for CKD followed by patient and provider education and (2) screen-educate plus a clinical pharmacist-led CKD and BP management program. Understanding the feasibility and value of pharmacist BP co-management among hypertensive persons with screen-detected CKD is important because BP management by pharmacists or nurses has been shown to be more effective than management by physicians alone [18–20].

This report describes our rationale for and design of the trial, as well as our experience in early implementation of the protocol. Communicating and disseminating the implementation realities of pragmatic trials is critical in CKD, as few of these studies are ongoing [21].

## Trial design and methods

### Trial overview

**Fig. 1:**
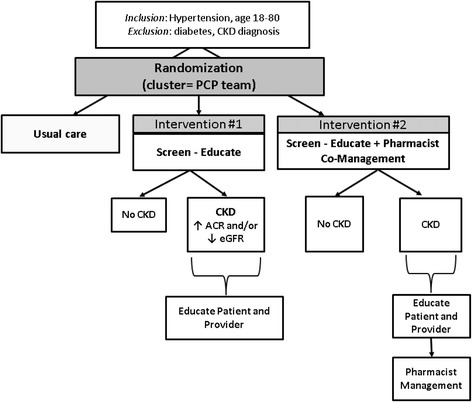
Study design

We designed the trial to have three groups: one *usual care arm*, and two intervention arms (the *screen & educate arm*, and the *screen & educate + pharmacist* arm) (Fig. 1).

The trial design takes advantage of the administrative data in the Veterans health Administration (VHA) electronic health record (EHR) to identify non-diabetic Veterans with hypertension who do not have a CKD diagnosis and who are patients of the San Francisco VA Health Care System (SFVAHCS) Medical Practice (MP) Clinic (Primary Care). Study staff order triple-marker labs (serum creatinine and cystatin C and urinary ACR) via the EHR for all study participants in the intervention arms who have an upcoming appointment with their primary care provider (PCP) in MP Clinic. PCPs receive the triple-marker screening results and each patient’s CKD status electronically to assist with categorizing each patient by eGFR and ACR. We also give each PCP appropriate, evidence-based treatment recommendations for CKD care with each triple-marker lab result. These are presented in the form of an electronic “research note” in the EHR that requires a signed acknowledgment by the PCP. The results are also mailed to patients in both intervention arms, along with information on CKD education materials developed by the National Kidney Disease Education Program (NKDEP). The screen & educate + pharmacist trial arm moves a step beyond the screen & educate arm. PCPs in this arm are encouraged to refer patients with screen-detected CKD to a primary care clinical pharmacist. The pharmacists use these appointments to address the use of ACE/ARB in persons with albuminuria, provide education on CKD, and recommend NSAID avoidance with a scripted visit.

### Objectives

Our aim was to address several gaps in knowledge that currently hinder the ability to reach consensus on the value of screening for CKD. First, we will determine the yield (new cases detected) from a CKD screening program using the triple-marker approach among non-diabetic hypertensive veterans in primary care. We will compare rates of creatinine, cystatin C and albuminuria testing and use of NSAIDs, ACE/ARB, and blood pressure (BP) levels across study arms. Among persons with *screen-detected CKD*, we will compare the *screen & educate arm* and *screen & educate & pharmacist* on the use of ACE/ARB in individuals with albuminuria, appropriate CKD documentation in the medical record, NSAID use, and BP levels. Finally, we will conduct a formal assessment of the program implementation using the Reach, Effectiveness, Adoption, Implementation, and Maintenance (RE-AIM) framework [22, 23] (Table 1).

**Table 1:** The six domains of the RE-AIM framework and the application to this trial

RE-AIM domain	Measure of interest	Ascertainment modality
Reach of target (individual level)	Identification of Patients for Inclusion and Exclusion Characteristics of enrolled vs. excluded or opt out Patient characteristics by arm Proportion who agree to participate	Research coordinator log Database query Chart review
Reach of target (setting/organization)	Acknowledgement of research note by PCP Result Letters Sent	Database Query Coordinator Log
Effectiveness (individual level)	Proportion Screened Yield of Testing Proportion who attend pharmacy visit BP level NSAID use ACE/ARB use CKD recognition	Questionnaire Database Query Pharmacist log Chart Review
Adoption by target (setting/organization level)	Perceived work flow Satisfaction with program	Structured Interviews Email Survey
Implementation (setting/organization level)	Fidelity monitoring Verify use of treatment algorithm by pharmacist	Structured interviews and questionnaire Pharmacist log
Maintenance/ Sustainability (provider)	Satisfaction with program	Email questionnaire
Maintenance/ Sustainability (patients)	BP control, use of NSAIDs, ACE/ARB after cessation of intervention	Database query
Resources	Time by pharmacist Nephrology consultations Costs	Time logs VA costs

### Setting

This study is being conducted within the primary care clinics at the SFVAHCS an urban, academically affiliated VA. These clinics include the medical practice (MP) and the women’s clinic (WC), which are the primary care clinics at the VA. We specifically designed this trial to function within the framework of a Patient Aligned Care Team Model (PACT), similar to a medical home model in which PCPs practice within the context of a team of allied health professionals including mental health, nursing, nutrition, social work and pharmacy. The VA is the largest health care system in US and the PACT model has been in place in the primary care clinics across the VA nationally since 2011. In particular, this study leverages the involvement of the pharmacist on the VA PACT team.

### Provider eligibility

There are two types of study subjects in this study: providers and patients. The attending PCPs are study subjects (and are also the unit of randomization). All PCPs (MDs and Nurse Practitioners) with active patient panels at SFVAMC were eligible to participate (*N* = 70 PCPs). We grouped the PCPs hierarchically by preceptor (attending) and resident medical practice (MP) “units.” Preceptors (attendings) practice independently, while residents are grouped in pairs for cross-over, and they are supervised by one of the attendings. A resident pair is considered one “unit” and each individual attending is also considered one unit or “team”. There were a total of 41 units/teams available for randomization. The trial was approved by the University of California Institutional Review Board. All attendings provided written informed consent to participate. While obtaining consent, PCPs were given a list of their eligible patients to facilitate exclusion procedures (see below). Pharmacists are also study subjects and also underwent informed consent procedures.

### Patient identification, eligibility and characterization

For patients, inclusion and exclusion from the trial happened in three distinct stages. First, we used administrative data from the VA Corporate Data Warehouse (CDW). Individual patients were considered eligible for CKD screening by this protocol and inclusion in the trial if they met all of the following criteria: age 18–80 years; had a visit with an eligible primary care provider at the SFVAMC during the18 months prior to 5/31/2015; had an outpatient diagnosis of hypertension (ICD-9-CM code 401.1 or 401.9 in Outpat_VDiagnosis) in the period 5/16/2010-5/15/2015; did not have an outpatient diagnosis of diabetes mellitus (ICD-9-CM code beginning “250.” in Outpat_VDiagnosis) for any date through 5/31/15; and did not have an outpatient diagnosis of chronic kidney disease (ICD-9-CM code beginning “585.” in Outpat_VDiagnosis) for any date through 5/31/15. We defined diagnosed CKD without consideration of creatinine or ACR in the laboratory section of the medical record, since work from our group and others has shown that awareness and recognition of CKD is extremely low, even among persons with documented reduced eGFR [14, 24]. We excluded persons who need specialty care: kidney transplant, pregnancy or eGFR < 15 ml/min/1.73 m^2^.

To further characterize patients, we assessed history of coronary artery disease (ICD-9-CM 410–414.99; ICD-10-CM I20-I25), congestive heart failure (ICD-9-CM 428.XX; ICD-10-CM I50.XX)], medication use based on fills at a VA pharmacy or paid for by VA, or non-VA medications reported by a patient, within six months prior to randomization, blood pressure levels within 1 year prior to randomization, outpatient laboratory data within 2 years prior to randomization (creatinine, urinary albumin) from VA electronic medical record data [CDW diagnoses, laboratory, non-VA medication, and vital status data, and Managerial Cost Accounting laboratory and pharmacy data].

To evaluate the fidelity of the EHR as data source for this study, we validated the inclusion criteria and three major relevant comorbidities: coronary artery disease, cerebrovascular disease and congestive heart failure with chart review. Two trained nephrologists (L.R. and S.V.) blinded to diagnoses obtained from the EHR reviewed 50 random charts to validate ICD-9-CM based definitions. A third reviewer (C.P.) assessed charts where there was disagreement. We used criteria as previously specified in a validated CKD registry [25].

### Additional patient exclusion procedures

PCPs had the option to exclude any patient that met exclusion criteria or who they deemed not appropriate for CKD screening. We provided PCPs a preliminary list of eligible patients and given the opportunity to exclude patients based on the criteria if life expectancy is <1 year or patients had a diagnosis of dementia, severe vision impairment, severe mental illness or substance abuse that significantly interferes with receiving care or inclusion in the study is not appropriate (e.g. due to cognitive dysfunction or severe anxiety). Patients were also excluded from the trial if they had heart failure identified by ICD-9-CM 428.0, and a reduced ejection fraction confirmed by echocardiography(<40%) within 5 years.

### Patient consent process

The study received waivers of informed consent and Health Insurance Portability and Accountability Act (HIPAA) authorization for obtaining limited data to identify patients for inclusion/exclusion. Following randomization of provider teams, eligible patients received a copy of the UCSF informed consent document and an information letter by mail, explaining the protocol and notifying them that orders for CKD screening would be submitted prior to their next scheduled appointment. Participants were given the option to opt-out of this study at any time by calling or mailing the card back. After the initial letter, an additional letter was sent to eligible patients of providers randomized to the usual care arm to clarify that they would remain eligible for CKD screening, by request, from their PCP. Participants who did not opt out by 6 weeks followed the mailing were included in the study.

### Randomization and blinding

Randomization occurred at the level of the MP or WC “unit” or “team” in order to avoid contamination across trial groups that may occur if patients in multiple arms are managed within the same team. We chose to block-randomize by number of patients per team and by attending vs. resident in order to balance these characteristics across study arms. Patients are linked to the PCP using the EHR, and were assigned to a trial arm based on their provider team’s assignment. PCPs are not blinded to treatment assignment after randomization since they facilitate the intervention arms. The analysts will remain blinded throughout the trial and data analyses.

### Description of study interventions

Prior to the beginning of this trial, all clinicians at the SFVAHCS had the opportunity for education on the international CKD guidelines in the form of a Grand Rounds. Clinicians were informed of this trial by the PI during provider staff meetings. All providers have access to educational materials such as UptoDate and the UCSF library.

#### Usual care

Following enrollment, PCPs randomized to usual care continue to manage the patients as usual. No systematic screening for CKD among non-diabetics is performed at SFVAMC. Referral to the PACT pharmacist remains an option, as this is part of usual care within the PACT model. Triple-marker screening for CKD is clinically available, and providers in the usual care arm are able to order these lab tests if deemed appropriate.

#### Intervention 1 (screen & educate)

We designed each intervention design to be feasible and to follow the usual workflow. For included patients, we identify their upcoming appointments using CDW data on a weekly basis. Study staff place orders for a serum creatinine, serum cystatin C and urinary albumin to creatinine ratio. All tests are performed locally at the clinical lab. Serum and urine creatinine are measured by the Jaffe method, cystatin C is measured using the particle-enhanced cystatin C assay from Gentian [26], and the urine albumin by nephelometry. Tests are ordered prior to the next scheduled visit with their assigned PCP. The study staff ensures that the PCP receives the test results, along with the CKD stage (or no CKD), and a summary of KDIGO guidelines for BP management appropriate for the specific CKD stage in the form of an electronic note which the PCP has to co-sign. These electronic notes also include a reminder to counsel on NSAID use, and the use of ACE/ARB in persons with albuminuria. (Additional file 1) All participating patients in the intervention arm receive notification of test results by mail. All persons with screen-detected CKD and their PCPs will receive educational materials available from the National Kidney Disease Education Program (NKDEP) as a web link on the note or letter they receive by mail.

#### Intervention 2 (screen-educate-active BP management by clinical pharmacist)

In addition to the screening and education as above, providers randomized to this arm will have the option to refer patients with screen-detected CKD to be co-managed by a clinical pharmacist. The clinical pharmacists at the VA hold doctoral degrees (PharmD) and they are active members of the care teams, as described above. The pharmacists already provide consultation for BP management at SFVAMC when referred by PCP. At SFVAMC, pharmacists are authorized to initiate therapy, adjust doses, monitor complications and order labs tests, under the supervision of the attending physician. Specific to this study, when the triple marker screen identifies CKD, research staff will notify providers through a note in the EHR that pharmacist co-management is available for their patient and they can refer to the pharmacist through the electronic consult request for CKD and hypertension management and education. Physicians in the other arms may still refer to pharmacy for other indications, but they are not notified of the availability of this service. Upon receipt of referral, the pharmacist schedules a series of appointments aimed at education on CKD and its complications, as well as education on avoiding NSAID use. The visit also focuses on BP control and initiation or up-titration of ACE/ARB therapy in persons with albuminuria. At each visit, the pharmacist measures and records two seated BP readings, and will perform medication inventory and review adherence. Education regarding diagnosis of CKD is based on previously designed materials provided by NKDEP that include lifestyle modification. Management follows evidence-based algorithms with the goal of achieving BP <140/90 mmHg and using ACE or ARBs for persons with albuminuria. Lower BP goals can be requested by the PCP. Initially, pharmacist follow-up visits are every 2 weeks, and once a patient’s BP is controlled on2 or more consecutive visits, follow-up ends. The pharmacist orders a chemistry panel to monitor for hyperkalemia or rising creatinine if ACE/ARB is initiated or uptitrated. If a patient is found to have eGFR <30 ml/min/1.73 m^2^, were commend nephrology referral for pre-dialysis care. At the end of 12 months, the pharmacist will stop following patients. To verify the fidelity of the educational intervention, the pharmacist presents several randomly chosen cases to the nephrologist involved in algorithm design. The pharmacists collaborated on creating the CKD visit algorithm along with study MDs (Additional file 2).

### Outcomes of interest

We will ascertain: (1) patient completion of ordered triple marker tests; (2) yield of testing, calculated as the proportion with new CKD cases identified among those tested; (3) acknowledgement of CKD results and CKD recognition by PCP; (4) use of ACE/ARB; (5) NSAID education and use, and (5) BP levels, estimated as change in blood pressure from enrollment to the end of the 12-month follow up period as a continuous outcome, from the CDW. The primary clinical outcome is systolic blood pressure level change from baseline. BP measures from the clinical record will be ascertained no more than quarterly. We will also consider the dichotomous outcome “achieved sustained BP control”, defined as BP <140/90 mmHg in ≥ two consecutive visits during the trial. The primary process outcome of this trial is triple marker screening using creatinine, cystatin C and ACR. Secondary process outcomes include CKD recognition and appropriate risk stratification by PCP, ACE/ARB use overall and in persons with albuminuria, NSAID use, NSAID education by PCP to patient. ACE or ARB use and NSAID use will be determined by ACE/ARB prescription from enrollment to 12-month follow-up, which will be ascertained from pharmacy data files quarterly. Documentation of CKD in EHR will be ascertained by using diagnostic codes and chart review. We will also compare CKD status at the end of the study using administrative codes vs. review of PCP notes. NSAID education from PCP to patient will be assessed by chart review. In exploratory analyses, we will record all follow up measures of serum creatinine, cystatin C and ACR in order to assess renal function decline or recovery.

### Sample size and power

All power calculations were performed using Stata version 11.2. Calculations were performed for 80% power assuming a two-tailed alpha level of 0.05 and intraclass correlation coefficient (ICC) of 0.025 to account for the clustering. We determined baseline rates/levels in usual care from CDW, and hypothesized that persons in the intervention arms would have lower BP levels and improved processes of care compared with usual care. For comparisons across the three arms, assuming approximately 450 persons per arm, for differences in systolic blood pressure (SBP) change, we will have 80% power to detect a difference ≥1.82 mmHg for SBP if we include 600 persons per arm, and ≥2.2 mmHg if we have 300 persons per arm. We powered the study for change in blood pressure levels.

## Results

### Reach of target at the patient level

#### Patient identification and participation

**Fig. 2:**
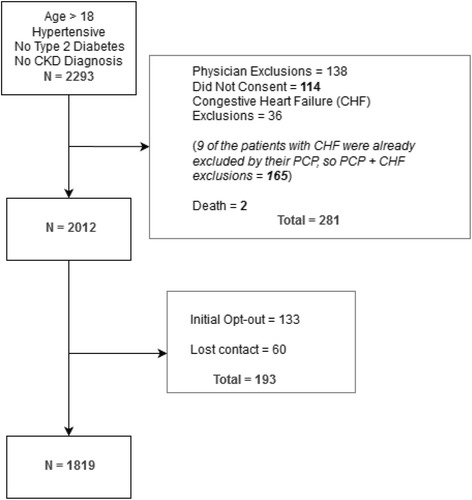
Consort diagram for patient inclusion

We identified 2,293 patients at the SFVAMC who met initial criteria. A total of 114 were either assigned to the provider who did not consent or to a study physician (M.S) and were thus excluded. Providers excluded an additional 138 patients after reviewing their patient list. We identified 36 patients with an EF < 40%, but 9 of these patients had already been excluded by their PCP, and 2 persons who had died since the data pull, and who had not previously been excluded by PCP. Since mailing of the information letters to 2012 patients, we received 141 telephone calls. Of these, 73 called to opt out. The rest of the calls reflected interest in the program and wanting to participate or alerting us to upcoming appointments. We have had only 1 phone call from a veteran stating that receiving the information letter caused great anxiety. We excluded a total of 193 persons who opted-out or had no available address and no other contact information. A total of 1,819 patients are included in this trial (Fig. 2).

The EHR administrative data accurately identified persons for inclusion and exclusion in this trial. Among 50 charts reviewed, one person had been living at the nursing home and had incomplete information to assess all the comorbidities. Of the remaining 49 charts, 45 (92%) had confirmed hypertension by the reviewers, and agreement for diabetes status was 100%. Agreement of EHR and physician review is shown in Table 2. While we found good agreement for coronary artery disease, we found less agreement for cerebrovascular disease and congestive heart failure, where chart review was more likely to identify these diagnoses that the EHR.

**Table 2:** Agreement between EHR and chart review for relevant comorbidities

	Chart Review			
	Overall Agreement	Kappa (95% CI)	PPA* (95% CI)	NPA* (95% CI)
Coronary Artery Disease	96%	0.883	91%	97%
		(0.724-1.000)	(86-100%)	(86-100%)
Cerebrovascular Disease	88%	0.188	20%	96%
		(-0.225-0.601)	(0.5-72%)	(84-99%)
Congestive Heart Failure	92%	0.456	50%	96%
		(0.006-0.905)	(7.0-93%)	(85-100%)

*PPA Positive Percent Agreement (mathematically equivalent to sensitivity), *NPA* Negative Percent Agreement (mathematically equivalent to specificity)

#### Participant characteristics

Overall, the median age (interquartile range) of 1, 819 included participants was 68 years (61–72), and only 8 were female. Approximately 16% are Black, and 5% Hispanic. We have found that 18% of participants have active prescription for NSAIDS, and 35% were on ACE/ARB at study start. We found that the majority of participants had a prior serum creatinine tested, and about 50% of participants had a prior urinary dipstick result in the chart. Less than 10% of persons had an albumin to creatinine ratio (ACR) tested prior to randomization. Characteristics of participants included in the study vs. those were excluded are presented in Table 3. Overall, excluded patients were similar in age, demographics, and comorbidities. Excluded persons were less likely to be using an anti-hypertensive medication, were somewhat less likely to have prior testing for proteinuria, and had a higher prevalence of drug use disorders. In the period between data pull and randomization, a total of *N* = 34 patients had new diagnoses of diabetes, and *N* = 11 had a new diagnosis of CKD among participant included in the study, but these were relatively evenly distributed by arm.

**Table 3:** Characteristics of veterans randomized, compared with those excluded

	Randomized (*N* = 1819)		Excluded (*N* = 193)		
Variable	N	(%)	N	(%)	*p*-value
Gender					0.877
Female	8	(0.4%)	1	(1%)	
Male	1811	(100%)	192	(99%)	
Age					0.242
26-50	169	(9%)	14	(7%)	
51-60	288	(16%)	22	(11%)	
61-70	805	(44%)	90	(47%)	
71-80	557	(31%)	67	(35%)	
Race/Ethnicity					0.575
White	1009	(55%)	104	(54%)	
Black	298	(16%)	27	(14%)	
Asian/Pacific Islander	150	(8%)	14	(7%)	
Amer. Indian	11	(1%)	1	(1%)	
Hispanic	92	(5%)	10	(5%)	
Missing	259	(14%)	37	(19%)	
CKD					0.477
No	1808	(99%)	191	(99%)	
Yes	11	(1%)	2	(1%)	
Ischemic Heart Disease	317	(17%)	37	(19%)	0.545
Congestive Heart Failure (core)	108	(6%)	7	(4%)	0.189
Cardiomyopathy	29	(2%)	1	(1%)	0.241
Cerebrovascular Disease	167	(9%)	13	(7%)	0.258
Diabetes Mellitus	34	(2%)	3	(2%)	0.757
Hyperlipidemia	1163	(64%)	124	(64%)	0.931
Hypertension (common)	1794	(99%)	191	(99%)	0.698
Chronic obstructive pulmonary disease and bronchiectasis	143	(8%)	12	(6%)	0.415
Tobacco Use Disorder	496	(27%)	48	(25%)	0.476
Drug Use Disorder	288	(16%)	42	(22%)	0.034
Alcohol Use Disorders	484	(27%)	46	(24%)	0.406
Mental Health Disorders	893	(49%)	91	(47%)	0.608
Any Hypertension Meds	1288	(71%)	116	(60%)	0.002
Beta Blockers	498	(27%)	37	(19%)	0.014
Ace Inhibitors	505	(28%)	43	(22%)	0.104
Angiotensin Receptor Blockers	134	(7%)	10	(5%)	0.263
Aspirin	250	(14%)	16	(8%)	0.033
Statins	793	(44%)	63	(33%)	0.003
NSAID	333	(18%)	25	(13%)	0.064
Urine – Dipstick Prior to Screening					<0.0005
Neg (<10 mg/dl)	810	(45%)	61	(32%)	
Trace (10-20 mg/dl	7	(0%)	0	(0%)	
1+ (30 mg/dl)	62	(3%)	15	(8%)	
2+ (100 mg/dl)	41	(2%)	6	(3%)	
3+ (300-500 mg/dl)	2	(0%)	2	(1%)	
Missing	897	(49%)	109	(56%)	
Albumin: Creatinine Ratio Tested	172	(9%)	7	(4%)	0.007
Creatinine Tested Prior	1658	(91%)	160	(83%)	<0.0005
Creatinine (mean, SD)	1.0	(0.2)	1.0	(0.2)	0.671
Cystatin C Tested Prior	83	(5%)	7	(4%)	0.550
Systolic Blood Pressure	139(18)		139(18)		0.87
Diastolic Blood Pressure	81(11)		81(9)		0.49

All Comorbidites were ascertained by the use of administrative data. Tests “prior” means done before trial started. SBP and DBP are ascertained as mean of values within 6 months prior to randomization date

### Reach of target at provider level

A total of 41 provider teams (representing 70 individual providers) were randomized. Two PCPs left the practice after they consented to participate and were randomized, but before testing was implemented, potentially affecting 179 patients. As per clinical workflow, these patients will be reassigned to an appropriate provider with availability, who may or may not have been randomized to the same trial arm as the original provider. In addition, 14 residents graduated, and these patients are expected to be reassigned to new trainees or existing providers. Changes in resident panels may affect 345 patients.

### Effectiveness in implementation: early experience in proportion screened and yield of testing

In the first 6 months of the trial, 434 discrete patients with appointments have been identified in the intervention arms, and orders for triple marker screening have been entered. Of these, 217(50%) have been tested as of September 15, 2016. We have identified 48 new CKD cases among those tested (22%). We have identified two cases of acute kidney injury.

### Early adoption

To date, we have not had any complaints from the providers about the study. A total of 217 research notes have been sent to PCP for co-signature. The P.I. has attended two staff meetings after protocol implementation to answer questions about the study.

One meeting with the pharmacists has occurred after protocol implementation to ensure fidelity of the pharmacy visit flow. All the participating pharmacists (3) were able to verify the study procedures at the meeting.

### Statistical analysis considerations based on implementation experience

The primary analyses will follow intention to treat principles. We have designated *a priori* sensitivity analyses will include “as treated” analyses, in order to understand the impact of patients changing physicians who may have been randomized to a different arm initially.

## Discussion

As CKD continues to represent a large burden in the U.S., understanding the value of systematic, early screening for CKD in targeted populations is paramount. In this report, we describe the rationale, design and initial implementation experience of a pilot pragmatic randomized trial to address this question. This report describes lessons learned from one of the few ongoing pragmatic randomized trials in the field of CKD. We have successfully implemented a protocol that uses the EHR to identify and characterize eligible participants, deliver the intervention, and ascertain study outcomes, consistent with the Institute of Medicine goals for a “learning health care system”. Our study is largely pragmatic, according to the PRECIS criteria for rating of trials [27]. We have also found that, thus far, there are high rates of participation by providers, the opt-out rate by patients was low, and a high proportion of patients with appointments have followed through with testing after the tests are ordered. Moreover, we learned several important “real-world” lessons that are likely to affect this study and design of future pragmatic trials in the field of CKD, including provider turnover, informed consent barriers, and data science considerations.

PCP and pharmacist participation in this study have been crucial. We believe that the frequent face-to-face communication between study staff and PCP prior to the study was an important step to ensure stakeholder engagement. The PI of this study (C.P.) had weekly meetings with the medical practice leadership during the planning phase to ensure the trial was designed to follow PCP workflow. We spent several weeks imbedded in the clinics to tell providers about the study and understand workflows. Since we took the time to gather accurate lists of their patients to be included and excluded, we reduced the amount of effort needed to identify patients who may not be appropriate for CKD screening or to participate in research. We engaged the pharmacists as stakeholders in building the intervention, which ensured it followed workflow, was feasible, and to ensure fidelity of the study procedures.

The finding that there is significant provider and patient turnover may affect the results of a pragmatic study randomized by team as it can lead to reduced protocol fidelity and contamination between arms. In the future, a larger pragmatic trial of CKD screening will need to randomize by clinic, rather than by provider to reduce contamination. In the case of this pilot, this was not feasible due to limited resources. Therefore, we have planned a priori analyses that will include intention-to-treat and as-treated. The ideal study would enroll patients on a “rolling” basis. For example, patients could be enrolled at the time they are identified as meeting inclusion criteria and having a scheduled appointment with the PCP. This strategy would allow the flexibility of changes in clinic assignments, and would ensure inclusion of patients still engaged with the system. This was not feasible in this study because we had to obtain informed consent via a mass mailing with a subsequent opt out period, making it very difficult to incorporate the many schedule changes that happen in clinical practice over this duration. We strongly advocate that pragmatic studies with very low risk interventions request a waiver of individual informed consent and perhaps only require that all patients are informed about the study and have the option to opt out by placing flyers in the clinic, or mailing short information pamphlets (rather than the full consent document) well before enrollment begins.

Finally, we would like to emphasize the critical importance of the data scientists in order to implement this EHR-based/reliant study successfully. We spent considerable time and resources building a custom database for study management. Moreover, an experienced data expert was necessary to obtain all the information from CDW and ensure accurate PCP assignment for each patient. The data scientists work as part of the team including the PI and the clinicians, in order to ensure accurate data use and to understand the clinical considerations. Future studies that rely on the EHR to deliver interventions must include a team with multi-disciplinary expertise and frequent meetings, and clinicians who can validate the data.

## Conclusions

In summary, we describe a successful implementation of a pilot RCT to evaluate the effectiveness of CKD screening to improve processes of care among hypertensive veterans without diabetes. Results from this study can guide design of pragmatic trials in the field of CKD.

## Additional files

Additional file 1: Sample doctor note. (DOCX 13 kb)

Additional file 2: Pharmacist visit flow chart. (DOCX 25 kb)

## Abbreviations

ACE/ARB: Renin-angiotensin system
ACR: Albumin-to-creatinine ratio
BP: Blood pressure
CDW: Corporate data warehouse
CKD: Chronic kidney disease
EF: Ejection fraction
eGFR: estimated glomerular filtration rate
eGFRcreat: estimated filtration rate by creatinine
EHR: Electronic health record
ESRD: End stage renal disease
HIPAA: Health insurance portability and accountability act
ICC: Intraclass correlation coefficient
KDIGO: Kidney disease improving global outcomes
MP: Medical practice (MP)
NKDEP: National kidney disease education program
NSAID: Non-steroidal anti-inflammatory drugs
PACT: Patient aligned care team model
PCP: Primary care physician
PRECIS: Pragmatic explanatory continuum indicator summary
RCT: Randomized clinical trial
RE-AIM: Reach, effectiveness, adoption, implementation, and maintenance
SBP: Systolic blood pressure
SFVAHCS: San Francisco VA health care system
UCSF: University of California, San Francisco
USPSTF: United States preventive services task force
VHA: Veterans health administration
