# Supplementary material for: Implementation of a pragmatic randomized trial of screening for chronic kidney disease to improve care among non-diabetic hypertensive veterans
Source: BMC Nephrol. 2017 Apr 12;18:132. doi: 10.1186/s12882-017-0541-6 (PMC5389143; doi:10.1186/s12882-017-0541-6)
Supplement: Supplementary file 1 — Sample doctor note. (DOCX 13 kb) [file 12882_2017_541_MOESM1_ESM.docx]

**Additional file 1**

**SAMPLE DOCTOR NOTE**

**SCREENING FOR CHRONIC KIDNEY DISEASE STUDY**

The results of the triple marker screen for CKD are as follows:

eGR by serum creatinine:

eGFR by serum cystatin C:

eGFR creat-cys combined:

Albumin to creatinine ratio (ACR):

**Guidelines suggest using a combined cystatin c/creatinine eGFR estimate. If the eGFR creatinine differs from the eGFR by cystatin C by > 30%, experts suggests using the cystatin c estimate**

*……………….If no CKD, note ends here……………….*

*If CKD is detected, continue:*

Your patient has chronic kidney disease (CKD+), stage …

**STEP # 1:** All patients with CKD should be appropriately staged using both eGFR and albuminuria. Consider adding CKD and stage to this patient's problem list.

For staging information refer to:

http://kdigo.org/home/guidelines/ckd-evaluation-management/

**STEP #2:** International guidelines recommend that, among persons with CKD:

- Blood pressure target should be <140/90 mmHg (use caution for age >75 years or frail elders)

- The first line agent for lowering BP in persons with albuminuria should be an ACE inhibitor or ARB. An anti-hypertensive should include a diuretic if needed for further BP lowering.

-Patient education including warning regarding caution using NSAIDs

Additional considerations: consider referral to nephrology if the eGFR is declining by >3ml/min/year, presence of hematuria, heavy proteinuria (>1 g per day), uncontrolled hypertension on at least 3 antihypertensive medications, or eGFR <30 ml/min/1.73m^2^.

*……….If randomized to screen-educate plus pharmacist…………*

**STEP #3**: This patient is eligible for a pharmacist consult to implement the CKD international guidelines outlined in STEP #2. If you would like your patient to see the pharmacist, please order a pharmacist consult for hypertension.
