# Supplementary material for: Implementation of a pragmatic randomized trial of screening for chronic kidney disease to improve care among non-diabetic hypertensive veterans
Source: BMC Nephrol. 2017 Apr 12;18:132. doi: 10.1186/s12882-017-0541-6 (PMC5389143; doi:10.1186/s12882-017-0541-6)
Supplement: Supplementary file 2 — Pharmacist visit flow chart. (DOCX 25 kb) [file 12882_2017_541_MOESM2_ESM.docx]

Additional file 2

**PHARMACIST VISIT FLOW CHART**

Step 2: Medication Reconciliation/Adherence Check

Step 6: CKD & NSAID education

Step 5: Referral to lab/dietitian as needed

Step 1: Patient Check-In

Step 4: Medication adjustment as per protocol

Step 3: Blood Pressure Check

**Appendix**

**Suggested Script for NSAID Education Recommendations During Pharmacy Visit**

- Nonsteroidal anti-inflammatory drugs, or NSAIDs, are over-the-counter pain relievers. Common types of NSAIDs are ibuprofen (Motrin, Advil) and naproxen (Aleve). There are more examples on the back of this sheet.
- People with diabetes and high blood pressure are at risk for kidney disease.
- NSAIDs may not be good for people at risk for kidney disease because they may cause bad kidney effects — such as lowering blood supply to the kidney.
- Adding NSAIDs to some blood pressure medicines can increase the possibility of having bad kidney effects, including lack of good blood flow to the kidneys.
- People with high blood pressure and/or diabetes should avoid NSAIDs even short term and should always contact their pharmacist or other care provider before they consider using an NSAID.
